# Disparities in Health-Related Internet Use Among African American Men, 2010

**DOI:** 10.5888/pcd11.130217

**Published:** 2014-03-20

**Authors:** Jamie A. Mitchell, Hayley S. Thompson, Daphne C. Watkins, Deirdre Shires, Charles S. Modlin

**Affiliations:** Author Affiliations: Hayley S. Thompson, Deirdre Shires, Wayne State University, Detroit, Michigan; Daphne C. Watkins, University of Michigan, Ann Arbor, Michigan; Charles S. Modlin Jr, The Cleveland Clinic Foundation, Cleveland, Ohio.

## Abstract

Given the benefits of health-related Internet use, we examined whether sociodemographic, medical, and access-related factors predicted this outcome among African American men, a population burdened with health disparities. African American men (n = 329) completed an anonymous survey at a community health fair in 2010; logistic regression was used to identify predictors. Only education (having attended some college or more) predicted health-related Internet use (*P* < .001). African American men may vary in how they prefer to receive health information; those with less education may need support to engage effectively with health-related Internet use.

## Objective

Patients who search for health information on the Internet and discuss their findings with physicians are more engaged during clinical encounters, better prepared to ask detailed questions (1), and more satisfied with their care (2).Those who engage in health-related Internet use tend to be white (3) and female (4,5). African American men are disproportionately burdened with health disparities (6,7), yet they are less likely to engage in health-related Internet use. Because patients who are more activated (ie, better equipped with the skills and confidence that enable active engagement in their own health care) during encounters with medical professionals have better health outcomes (8), a lack of health-related Internet use may contribute to health disparities. On the basis of previous studies (4,5), we hypothesized that socioeconomic factors and health care access may explain differences in health-related Internet use among African American men.

## Methods

We conducted a survey of African American men attending an annual community health fair hosted by the Cleveland Clinic Foundation in 2010. The fair provided free health screenings such as blood pressure and diabetes testing. The 329 participants were 18 years or older, could read and write in English, and verbally agreed to complete an anonymous 47-item survey that took approximately 10 minutes to complete. Study participants constituted 27% of all health fair attendees. Student volunteers administered the surveys; the institutional review board at the Cleveland Clinic approved this study. Behavioral measures were questions from previously validated instruments used in nationally representative health studies.

The dichotomous outcome measure, health-related Internet use, was assessed by asking participants to identify their most recent source of health information. The Internet was coded as 1; all other sources were coded as 0. All predictors were also coded as dichotomous, including age (≥65 vs <65); education (some college or more vs high school diploma or less); income (annual income <$25,000 vs ≥$25,000); chronic illness (diagnosis of heart disease, hypertension, diabetes, kidney disease, or high cholesterol vs none); sick leave (sick leave available from most recent or previous employment vs not available); identification of usual source of care (≥1 health care provider identified vs none); and emergency department (ED) use (ED as most frequent care source vs not most frequent).

Descriptive analyses examined sociodemographic and health characteristics. A χ^2^ test identified factors associated with health-related Internet use, and hierarchical logistic regression determined significant predictors of health-related Internet use. Analyses were conducted using SPSS 19 (IBM Corporation, Armonk, New York).

## Results

The mean age of participants was 49 (standard deviation, 12.2 y); 53% had attended at least some college; 37.4% were married; 57.4% were employed; 45% reported an annual household income of under $25,000; 47% reported having at least 1 chronic illness; and 26% used the ED as their most frequent source of health care. Only 31% had sick leave available from their previous or most recent employer. The Internet was the most recent source of health information for 39% of participants.

Cross-tabulations of the outcome variable with potential predictors showed that education, income, chronic illness, sick leave, and ED use were significantly associated with health-related Internet use (Table 1). Age and usual source of care were not significant. Participants with some college education or more, those with an annual income of over $25,000, those with a diagnosis of at least 1 chronic illness, those with sick leave available, and those who did not report the ED as their most frequent care source were more likely to have used the Internet as their most recent source of health information.

**Table 1:** Cross-Tabulations of Health-Related Internet Use and Sociodemographic, Medical, and Health-Care–Access Factors Among African American Men (n = 329) Surveyed at a Community Health Fair, 2010

Factor	Most Recent Source of Health Information Was Internet, %	Other Source of Information Used, %	χ^2^ (*P* Value)
**Age, y**			
<65	39.5	60.5	1.3 (.25)
≥65	28.6	71.4	
**Education**			
High school diploma or less	20.1	79.9	48.7 (<.001)
Some college or more	54.9	45.1	
**Annual income, $**			
<25,000	28.4	71.6	11.9 (.001)
≥25,000	47.0	53.0	
**Chronic illnesses**			
None	32.2	67.8	6.4 (.01)
≥1	45.8	54.2	
**Sick leave available**			
No	32.0	68.0	13.6 (<.001)
Yes	53.5	46.5	
**Identification of usual source of care**			
None	38.9	61.1	.003 (.96)
≥1 provider	38.6	61.4	
**Emergency department use**			
Most frequent health care source	29.1	70.9	4.5 (.04)
Not most frequent health care source	42.0	58.0	

Only education (having attended some college or more) significantly predicted health-related Internet use in the final regression model (Table 2). When other factors were controlled, African American men with some college education or more were nearly 4 times as likely to report recent health-related Internet use than African American men with a high school diploma or less (OR = 3.9; 95% CI, 2.3–6.5). The predicted probability of health-related Internet use was .55 for participants with some college education or more and .20 for those with a high school diploma or less.

**Table 2:** Sequential Logistic Regression Models of the Relationship Between Health-Related Internet Use and Selected Factors Among African American Men (n = 329) Surveyed at a Community Health Fair, 2010

Model	OR (95% CI)	*P* Value
**Model 1^a^**		
Education	4.33 (2.6–7.2)	<.001
Income	0.60 (0.4–1.0)	.046
**Model 2^b^**		
Education	4.16 (2.5–6.9)	<.001
Income	0.62 (0.4–1.0)	.048
Chronic illness	1.56 (1.0–2.5)	.07
**Model 3^c^**		
Education	3.90 (2.3–6.5)	<.001
Income	0.73 (0.4–1.3)	.25
Chronic illness	1.49 (0.9–2.4)	.11
Sick leave	1.54 (0.9–2.7)	.13
Emergency department use	0.85 (0.5–1.5)	.59

Abbreviations: OR, odds ratio; CI, confidence interval.

a Constant = 0.33; *P* < .001; R^2^ = 0.18.

b Constant = 0.24; *P* < .001; R^2^ = 0.19.

c Constant = 0.24; *P* < .001; R^2^ = 0.20.

## Discussion

This study addresses a gap in knowledge on disparities in health-related Internet use among African American men. Health-related Internet use is common among this group; almost 40% of respondents reported that the Internet was their most recent source of health information. Contrary to evidence that suggests that patients lacking health insurance or convenient health care providers may be more likely to engage in health-related Internet use (5), we found no relationship between health-related Internet use and health care access. Education level was the only significant predictor; as others have found (4), men with higher education levels were more likely to engage in health-related Internet use.

These findings have several implications for health promotion and practice. First, health providers should note that African American men may vary in how they prefer to receive health information. Others have found that this population is less likely to talk to providers about health-related Internet use but more likely to use the health-related information on Internet to change their health behavior (9). Therefore, health providers may need to actively engage African American men in discussions about health-related Internet use. Second, African American men with lower education levels may need support for increasing their engagement in health-related Internet use — a goal consistent with *Healthy People 2020* (10). Such support may include promoting reliable health-related websites and assisting African American male patients — regardless of education level — in assessing the quality of information they have found.

Limitations of this study include a pilot sample size and the use of convenience sampling. However, our sample is demographically similar to nationally representative samples of African American men (11) in terms of marital status, education level, income, and unemployment. Future studies should assess ways to increase health-related Internet use among African American men with lower education levels as well as determine how health outcomes are affected by health-related Internet use among this population.

## References

[1] Bass SB , Ruzek SB , Gordon TF , Fleisher L , McKeown-Conn N , Moore D . Relationship of internet health information use with patient behavior and self-efficacy: experiences of newly diagnosed cancer patients who contact the National Cancer Institute’s Cancer Information Service. J Health Commun 2006;11(2):219–36. 10.1080/10810730500526794 16537289

[2] Hay MC , Cadigan JR , Khanna D , Strathmann C , Lieber E , Altman R , Prepared patients: internet information seeking by new rheumatology patients. Arthritis Rheum 2008;59(4):575–82. 10.1002/art.23533 18383399

[3] Fox S , Jones S . The social life of health information Washington (DC): Pew Internet and American Life Project; 2009. http://www.pewinternet.org/Experts/~/link.aspx?_id=62F4D7EFB49C4F9FA384FDC9D3A4B49B&_z=z. Accessed May 1, 2013.

[4] Atkinson NL , Saperstein LS , Pleis J . Using the internet for health-related activities: findings from a national probability sample. J Med Internet Res 2009;11(1):e4. 10.2196/jmir.1035 19275980 PMC2762768

[5] Bundorf MK , Wagner TH , Singer SJ , Baker LC . Who searches the internet for health information? Health Serv Res 2006;41(3 Pt 1):819–36. 10.1111/j.1475-6773.2006.00510.x 16704514 PMC1713205

[6] Mitchell J . Examining the influence of social ecological factors on prostate cancer screening in urban African-American men. Soc Work Health Care 2011;50(8):639–55. 10.1080/00981389.2011.589891 21919642

[7] Modlin CS . Culture, race, and disparities in health care. Cleve Clin J Med 2003;70(4):283,287–8. 10.3949/ccjm.70.4.283 12701982

[8] Hibbard JH , Greene J . What the evidence shows about patient activation: better health outcomes and care experiences; fewer data on costs. Health Aff (Millwood) 2013;32(2):207–14. 10.1377/hlthaff.2012.1061 23381511

[9] Rooks RN , Wiltshire JC , Elder K , BeLue R , Gary LC . Health information seeking and use outside of the medical encounter: is it associated with race and ethnicity? Soc Sci Med 2012;74(2):176–84. 10.1016/j.socscimed.2011.09.040 22154611

[10] US Department of Health and Human Services. Healthy People 2020. http://www.healthypeople.gov/2020/topicsobjectives2020/default.aspx. Accessed May 1, 2013. 10.3109/15360288.2015.1037530 26095483

[11] DeNavas-Walt C , Proctor BD , Smith JC . Income, poverty, and health insurance coverage in the United States: 2009. Washington (DC): US Census Bureau. http://www.census.gov/prod/2010pubs/p60-238.pdf. Accessed February 7, 2014.

